# Invalids

**Published:** 1878-05

**Authors:** 


### *INVALIDS.*

It is a discouraging fact that a majority of invalids have been made so through their own neglect or ignorance, or, in the cases of children, through the neglect or ignorance of their parents. The advance in medical science has done much to increase the average term of human life, but it is doubtful if it has greatly lessened the number of perpetual invalids, because of the almost universal disregard for its teachings and its warnings. There are certain unnatural conditions of the system that occasion more or less inconvenience, but are unheeded by the man intent on business or pleasure—that inevitably lead to suffering and to premature death unless promptly and intelligently treated and cured.

But in very many cases competent medical aid is not sought until organic changes have made a permanent cure impossible. We will again repeat what we have so often insisted upon, that *constipation* is the cause of half the chronic diseases that exist. And, still, there are comparatively few, even among intelligent people, who realize that it is a most unfortunate complaint, or who make any persistent effort to rid themselves of it. They even neglect the health and future happiness of their children, by neglecting to inform themselves of their physical condition and habits, and thus become morally responsible for the suffering they might easily have prevented.

Purgatives should not be habitually used; but a natural state of the bowels should be secured by the local method devised by M. Nelaton, the celebrated French Surgeon. His 'Suppository" is now generally employed by European physicians in lieu of purgative medicines.

